# Bacterial biota associated with the invasive insect pest *Tuta absoluta* (Meyrick)

**DOI:** 10.1038/s41598-024-58753-w

**Published:** 2024-04-09

**Authors:** A. A. Lateef, A. A. Azeez, W. Ren, H. S. Hamisu, O. A. Oke, F. O. Asiegbu

**Affiliations:** 1 Department of Forest Sciences, University of Helsinki, Helsinki, Finland; 2 Department of Plant Biology, University of Ilorin, Kwara State, Ilorin, Nigeria; 3 National Horticultural Research Institute, Ibadan, Nigeria; 4 Rainforest Research Station, Forestry Research Institute of Nigeria, Jericho Hill, Ibadan, Nigeria

**Keywords:** Microbiology, Molecular biology, Ecology

## Abstract

*Tuta absoluta* (the tomato pinworm) is an invasive insect pest with a highly damaging effect on tomatoes causing between 80 and 100% yield losses if left uncontrolled. Resistance to chemical pesticides have been reported in some *T. absoluta* populations. Insect microbiome plays an important role in the behavior, physiology, and survivability of their host. In a bid to explore and develop an alternative control method, the associated microbiome of this insect was studied. In this study, we unraveled the bacterial biota of *T. absoluta* larvae and adults by sequencing and analyzing the 16S rRNA V3-V4 gene regions using Illumina NovaSeq PE250. Out of 2,092,015 amplicon sequence variants (ASVs) recovered from 30 samples (15 larvae and 15 adults), 1,268,810 and 823,205 ASVs were obtained from the larvae and adults, respectively. A total of 433 bacterial genera were shared between the adults and larval samples while 264 and 139 genera were unique to the larvae and adults, respectively. Amplicon metagenomic analyses of the sequences showed the dominance of the phylum Proteobacteria in the adult samples while Firmicutes and Proteobacteria dominated in the larval samples. Linear discriminant analysis effect size (LEfSe) comparison revealed the genera *Pseudomonas, Delftia* and *Ralstonia* to be differentially enriched in the adult samples while *Enterococcus*, *Enterobacter*, *Lactococcus*, *Klebsiella* and *Wiessella* were differentially abundant in the larvae. The diversity indices showed that the bacterial communities were not different between the insect samples collected from different geographical regions. However, the bacterial communities significantly differed based on the sample type between larvae and adults. A co-occurrence network of significantly correlated taxa revealed a strong interaction between the microbial communities. The functional analysis of the microbiome using FAPROTAX showed that denitrification, arsenite oxidation, methylotrophy and methanotrophy as the active functional groups of the adult and larvae microbiomes. Our results have revealed the core taxonomic, functional, and interacting microbiota of *T. absoluta* and these indicate that the larvae and adults harbor a similar but transitory set of bacteria. The results provide a novel insight and a basis for exploring microbiome-based biocontrol strategy for this invasive insect pest as well as the ecological significance of some of the identified microbiota is discussed.

## Introduction

On a global scale, insect pests and pathogens are one of the major constraints in crop production^1^ with up to $2.5 billion per year as cost for their control^2^. *T. absoluta*, also called the Tomato pinworm or leafminer, is a destructive invasive insect pest that originated from South America and has spread to other regions of the world including Europe, the Middle East and Africa where the damage is increasingly occurring^3,4^. It primarily infests tomato plants both in greenhouse and in the open field^5^, but can also invade other economic crops such as pepper and eggplant^6^. *T. absoluta* is a significant threat to tomato farmers worldwide, which if left uncontrolled can cause severe losses of up to 100%^4^.

Chemical control is usually the main method for control of *T. absoluta* in several countries and recently with biological control agents like nematodes^4,7^. However, the use of synthetic pesticide products often results in serious environmental degradation and economic issues, as a result, food and environment are contaminated, cost of production driven up and natural enemies of the insect pests are killed. In addition, some population of *T. absoluta* have the ability to develop rapid resistance to these chemicals^8–11^. Also, the effectiveness of synthetic pesticides is limited as the larvae are difficult to target with insecticide sprays due to their endophytic feeding behaviour^12^. *T. absoluta* belongs to the insect order Lepidoptera which undergo complete metamorphosis (holometabolous) with their larval stage being the period of active feeding and growth^13,14^.

Alternative control strategies should therefore be used within the context of integrated pest management (IPM) for this destructive pest. In the field of pest management, the microbiome of insects has emerged as a promising tool with immense potential for enhancing insect biocontrol strategies^15–18^. By understanding the complex interactions between insect hosts and their associated microbes, scientists are uncovering new ways to control pests and reduce the reliance on traditional chemical pesticides^19^. The understanding and manipulation of the microbial communities associated with insects as a means of biocontrol has proven to be effective in managing pest populations and maintaining ecological balance in agricultural systems^20^. Furthermore, targeting the microbiome directly or altering it to reduce the vector competence of pests can have significant implications in managing insect populations^15^.

The microbiomes associated with insect pests are highly diverse. The dynamics within and between members of this microbial communities which include fungi, bacteria and viruses affect the fitness and behaviour of insect pests in various ways^21^. Studies on insect pest microbiota have demonstrated that various factors shape the microbial community composition, ranging from life stages to the environment as well as host genetics^22^. In some cases, microbes also contribute towards the pest status of invasive insects^23^.

Knowledge about the insect microbiome can be harnessed to control invasive insect pests^24^. Most studies of the lepidopteran microbiota and specifically few studies on *T. absoluta* have focused only on the adult stage which only reveal a snapshot of the microbial community at that stage. Little is known about the changes in the microbiome at the most important stages of larvae. Previous research on the amplicon metagenomic analyses of the microbiome of invasive insect pest have revealed core microbiota associated with their host. Earlier studies^25,26^ on larvae of Fall armyworm (*Spodoptera frugiperda*), causing destruction in maize have revealed the dominance of the phyla Actinobacteria, Proteobacteria and Firmicutes. Similar trend was also recorded on adult *T. absoluta* in China^27^ with the phyla Proteobacteria and Firmicutes dominating the microbiome and with samples from different regions having similar microbiome structures.

However, little is known about the complex microbial community associated with both adult and larvae of *T. absoluta* particularly in sub-saharan Africa. In the present study, we used the NovaSeq Illumina NGS platform to reveal the bacterial microbiome of the larvae and adults of *T. absoluta*, the network interaction and potential ecological functions.

## Materials and methods

### Sample collection

Larval and adult samples of *T. absoluta* were randomly collected from farmers’ tomato fields based on availability. The sampling was done from August to October during the 2022 farming season from six different locations within two agroecological zones in Nigeria: North Central and North West (Table 1).

**Table 1:** The sample collection locations and their coordinates.

S/N	Sample ID	Sample type	Location	Region	GPS
1	F1	Larvae	Ilorin	Northcentral	8° 26′ 23.8848″ N, 4° 31′ 28.2144″ E
2	G1	Larvae	Ilorin	Northcentral	8° 26′ 23.8848″ N, 4° 31′ 28.2144″ E
3	H1	Larvae	Ilorin	Northcentral	8° 26′ 23.8848″ N, 4° 31′ 28.2144″ E
4	J1	Larvae	Ilorin	Northcentral	8° 26′ 23.8848″ N, 4° 31′ 28.2144″ E
5	K1	Larvae	Ilorin	Northcentral	8° 26′ 23.8848″ N, 4° 31′ 28.2144″ E
6	Q1	Larvae	Minna	Northcentral	9° 34′ 44.5254″ N, 6° 33′ 15.6852″ E
7	R1	Larvae	Minna	Northcentral	9° 34′ 44.5254″ N, 6° 33′ 15.6852″ E
8	S1	Larvae	Minna	Northcentral	9° 34′ 44.5254″ N, 6° 33′ 15.6852″ E
9	T1	Larvae	Minna	Northcentral	9° 34′ 44.5254″ N, 6° 33′ 15.6852″ E
10	U1	Larvae	Minna	Northcentral	9° 34′ 44.5254″ N, 6° 33′ 15.6852″ E
11	V1	Larvae	Kano	Northwest	11° 58′ 31.458″ N, 8° 32′ 47.1114″ E
12	W1	Larvae	Kano	Northwest	11° 58′ 31.458″ N, 8° 32′ 47.1114″ E
13	X1	Larvae	Kano	Northwest	11° 58′ 31.458″ N, 8° 32′ 47.1114″ E
14	Y1	Larvae	Kano	Northwest	11° 58′ 31.458″ N, 8° 32′ 47.1114″ E
15	Z1	Larvae	Kano	Northwest	11° 58′ 31.458″ N, 8° 32′ 47.1114″ E
16	AA1	Adults	Bagwai, Kano	Northwest	12° 9′ 11.56″ N, 8° 8′ 14.49″ E
17	AB1	Adults	Bagwai, Kano	Northwest	12° 9′ 11.56″ N, 8° 8′ 14.49″ E
18	AC1	Adults	Bagwai, Kano	Northwest	12° 9′ 11.56″ N, 8° 8′ 14.49″ E
19	AD1	Adults	Bagwai, Kano	Northwest	12° 9′ 11.56″ N, 8° 8′ 14.49″ E
20	AE1	Adults	Bagwai, Kano	Northwest	12° 9′ 11.56″ N, 8° 8′ 14.49″ E
21	AF1	Adults	Kazaure, Jigawa	Northwest	12° 38′ 58.956″ N, 8° 24′ 51.8034″ E
22	AG1	Adults	Kazaure, Jigawa	Northwest	12° 38′ 58.956″ N, 8° 24′ 51.8034″ E
23	AH1	Adults	Kazaure, Jigawa	Northwest	12° 38′ 58.956″ N, 8° 24′ 51.8034″ E
24	AI1	Adults	Kazaure, Jigawa	Northwest	12° 38′ 58.956″ N, 8° 24′ 51.8034″ E
25	AJ1	Adults	Kazaure, Jigawa	Northwest	12° 38′ 58.956″ N, 8° 24′ 51.8034″ E
26	AK1	Adults	Rogo, Kano	Northwest	11° 34′ 0.00″ N, 7° 49′ 60.00″ E
27	AL1	Adults	Rogo, Kano	Northwest	11° 34′ 0.00″ N, 7° 49′ 60.00″ E
28	AM1	Adults	Rogo, Kano	Northwest	11° 34′ 0.00″ N, 7° 49′ 60.00″ E
29	AN1	Adults	Rogo, Kano	Northwest	11° 34′ 0.00″ N, 7° 49′ 60.00″ E
30	AO1	Adults	Rogo, Kano	Northwest	11° 34′ 0.00″ N, 7° 49′ 60.00″ E

The North Central (Ilorin and Minna collection points) is a Southern Guinea Savannah with a mean annual rainfall of 1150 mm^30^, while the North West (Jigawa and Kano), a Sudan Savannah, has a mean annual rainfall of 1000 mm^31^. Larval samples were collected from the leaves and upper stem by hand picking while adults were collected by swipe net. The adult and larval samples were stored in 90% ethanol in 15 mL falcon and 2 mL Eppendorf tubes, respectively. DNA extraction for all samples was done at the Forest Pathology Research Lab at the University of Helsinki, Finland.

### DNA extraction, polymerase chain reaction (PCR), amplification of 16S region and Illumina next generation sequencing

Total genomic DNA was extracted from the larvae and adults of *T. absoluta* using a modified cetyltrimethylammonium bromide (CTAB) protocol^32^. Briefly, the protocol is as follows, the larval and adult samples were grinded with a micropestle in a 2 ml tube in presence of liquid nitrogen. CTAB extraction buffer (pre-heated at 65 °C) and DTT (1 mol/L) was added to the tube, vortexed and incubated for 30 min at 65 °C on an Eppendorf Thermomixer Comfort (900 rpm). One volume of chloroform:isoamylalcohol (24:1) was added to the tube, mixed together and then centrifuged at 10,000*g* for 10 min, after which the upper phase (about 700 μL) was transferred to a new tube. Subsequently, another one volume of chloroform:isoamyl alcohol (24:1) was added to the tube, mixed and centrifuged at 10,000*g* for 10 min. The supernatant was then transferred into a new 1.5 ml Eppendorf tube, 40 μL NaAc (3 M) and one volume of cold isopropanol were added and incubated at − 20 °C for 20 min. The tube was then centrifuged at 10,000*g* for 20 min at 4 °C. The supernatant was discarded, and the pellet was washed with 70% ethanol at 12,000*g* for 5 min at 4 °C. Thereafter, the pellet was dried by discarding the supernatant and inverting the tube in a sterile hood. The DNA was then resuspended in Milli-Q water. The DNA extracted was quantified using NanoDrop Spectrophotometer (2000C, Thermo Scientific) following the manufacturer's protocol.

PCR amplification of the V3–V4 regions of the 16S rRNA regions were performed using the primers 341F (CCTAYGGGRBGCASCAG) and 806R (GGACTACNNGGGTATCTAAT)^33^ connecting with barcodes. The PCR products with proper size were selected by 2% agarose gel electrophoresis. Libraries were made by pooling equal amounts of PCR products from each sample, end-repaired, A-tailed, and then ligated with Illumina adapters. Libraries were sequenced on a paired-end Illumina platform at Novogene (UK) to generate 250 bp paired-end raw reads (Raw PE). The raw reads were demultiplexed, adapters trimmed and raw data with the sequencing quality information were recorded in a FASTQ file^34^.

### Processing of sequencing data

The paired-end reads were demultiplexed and assigned to samples based on their unique barcodes. Barcodes and primer sequences were cut off from the reads. Quality filtering of the raw reads was performed under specific filtering conditions to obtain the high-quality clean reads. Paired-end reads for each sample were merged using FLASH Version 1.2.11 (http://ccb.jhu.edu/software/FLASH/)^35^ and then spliced when reads overlapped. The paired-end sequence data were further processed through the Quantitative Insights Into Microbial Ecology (QIIME2) pipeline^36^. Reads were denoised (i.e. filtered and dereplicated) based on quality scores (below Q30), chimera were detected and removed using the DADA2 algorithm^37^ to generate amplicon sequence variants (ASVs).

### Amplicon sequence variant (ASV) assignment and taxonomic annotation

Amplicon sequence variants (ASVs) taxonomic assignments were done against a pre-trained Naive Bayes classifier and QIIME2-compatible SSUrRNA SILVA database (clustering at 97% similarity threshold, release 138 (http://www.arb-silva.de/)^38,39^ at each taxonomic rank. Abundance normalization of the ASVs were done based on the smallest sample size (31,101) and was used in further analysis. Rarefaction and diversity analyses including ACE^40^, Chao1^41^, Shannon and Simpson indices^42^ were done both in Qiime2^43^ and in Rstudio (version 4.2.0). Principal coordinate analysis (PCoA) was used to assess the Beta diversity with the weighted UniFrac (Bray–Curtis) distance matrix of the relative abundance of ASVs calculated by the QIIME2 using the vegan and ggplot2^44^ package in RStudio (version 4.2.0)^45,46^. Further analyses and visualizations were carried out using R packages which include qiime2R (v0.99.6) (https://github.com/jbisanz/qiime2R?search=1), phyloseq (v1.38.0)^47^, and tidyverse (v2.0.0)^48^.

### Statistical analyses of data

Differences in the alpha diversity between samples and relative genera abundances were tested using Wilcoxon rank-sum and Kruskal–Wallis tests (with post hoc Dunn test). Permutational analysis of variance (PERMANOVA) and Kruskal–Wallis test (at p ≤ 0.05) were used to compare the differences in the beta diversity. Microbial ecological network analyses^49^ was done to assess the interaction between each taxa based on the ASV abundance correlations using the psych package in R and visualized in Gephi (Version 0.9.2). Functional annotation of prokaryotic taxa (FAPROTAX) was used to predict the ecological functional groups of the taxa using the annotated prokaryotic 16S sequence database^50,51^.

### Deposition of nucleotide sequences

The sequences obtained in this study were deposited in the GenBank short-read archive (SRA) with accession number PRJNA1032433 (https://dataview.ncbi.nlm.nih.gov/object/PRJNA1032433).

## Results

### Sequencing data

A total of 2,561,953 paired-read sequences were obtained from 30 samples which after quality filtering, denoising and chimera removal yielded 2,092,015 sequences (Supplementary Table S1). The sequences from each sample ranged from 31,101 to 102,707 sequences with an average ± standard deviation of 71,276 ± 20,000 reads per sample.

**Figure 1:**
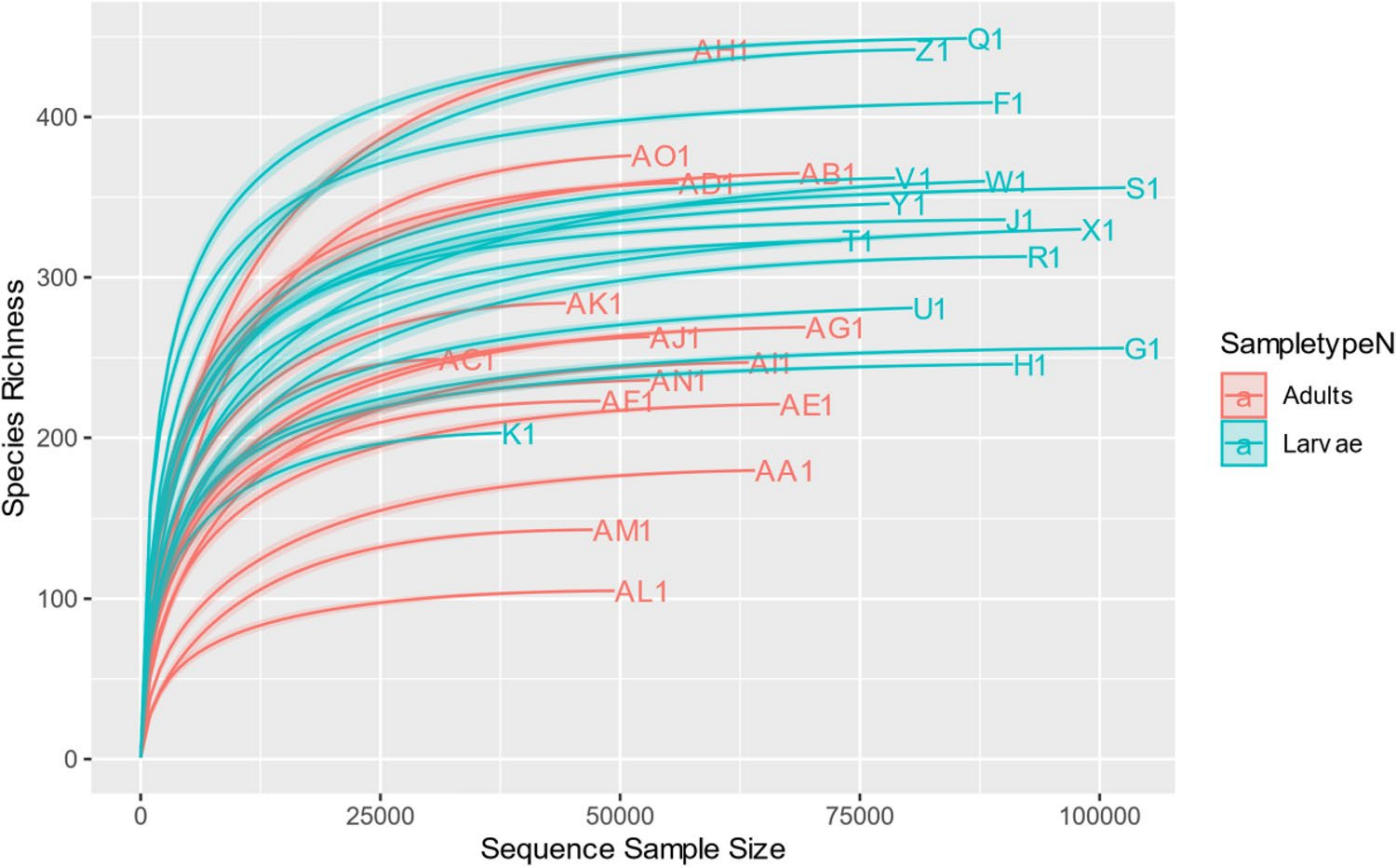
Rarefaction curves of the 30 samples.

All samples had good coverage based on the rarefaction curves (Fig. 1).

From all the 2,092,015 ASVs recovered from the 30 samples (15 larvae & 15 adults), 1,268,810 ASVs were obtained from the larvae and 823,205 ASVs from the adults. All the ASVs in all samples were identified to belong to bacteria (99.8%) with only 1 sample (AO1) containing an unassigned proportion of 6.86% (Supplementary Fig. S1).

### Composition and structure of *T. absoluta* bacterial biota

**Figure 2:**
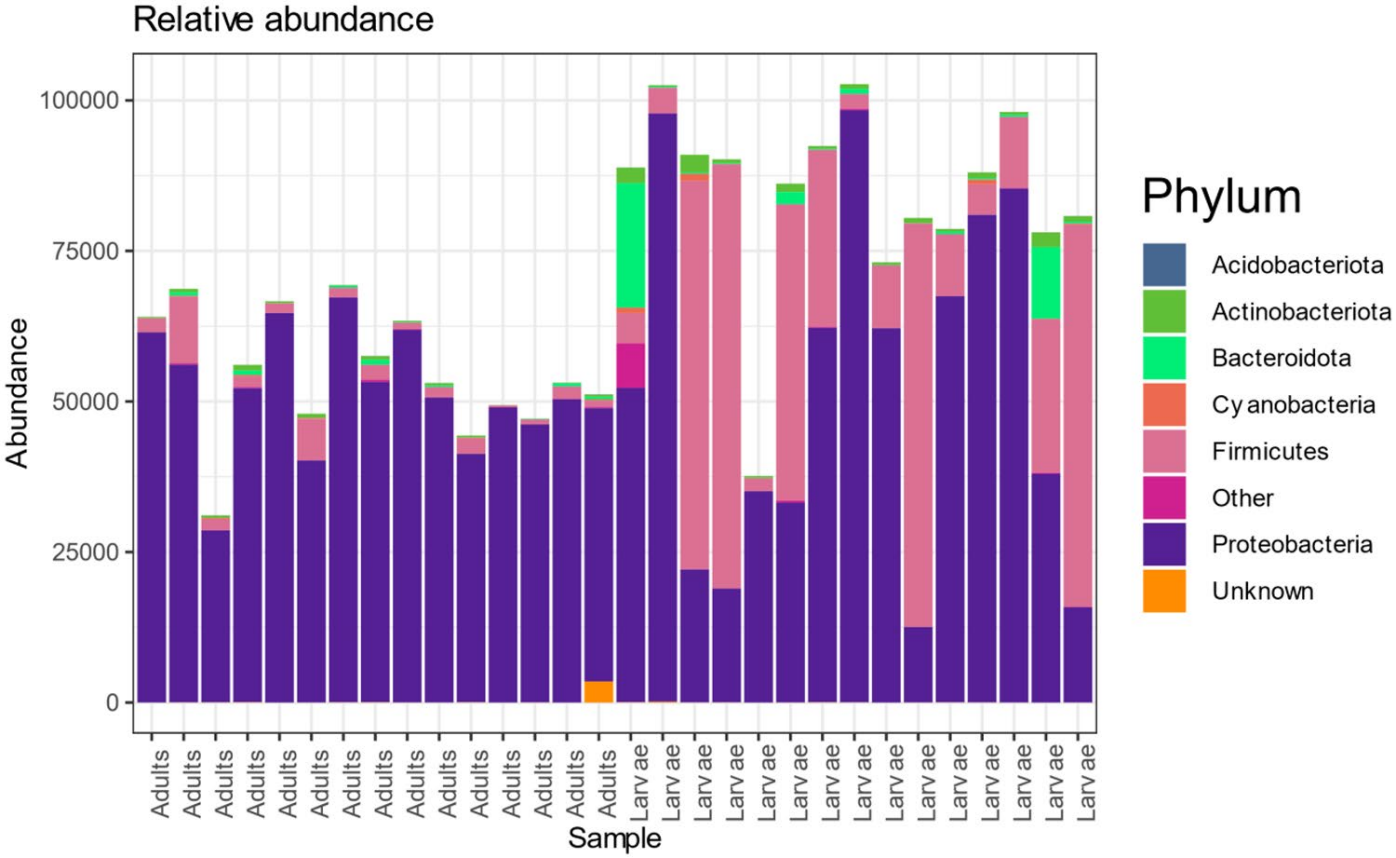
Top 10 bacteria phyla from the adult and larval samples.

The bacterial biota was classified into 36 phyla with Proteobacteria (77.56%) being the most abundant followed by Firmicutes (18.86%), Bacteroidota (1.71%), Actinobacteria (0.96%), Bdellovibrionota (0.27%) and Cyanobacteria (0.22%). The phyla Proteobacteria were more abundant in the adult sample than in the larvae and both Proteobacteria and Firmicutes were more abundant in the larvae (Fig. 2).

**Figure 3:**
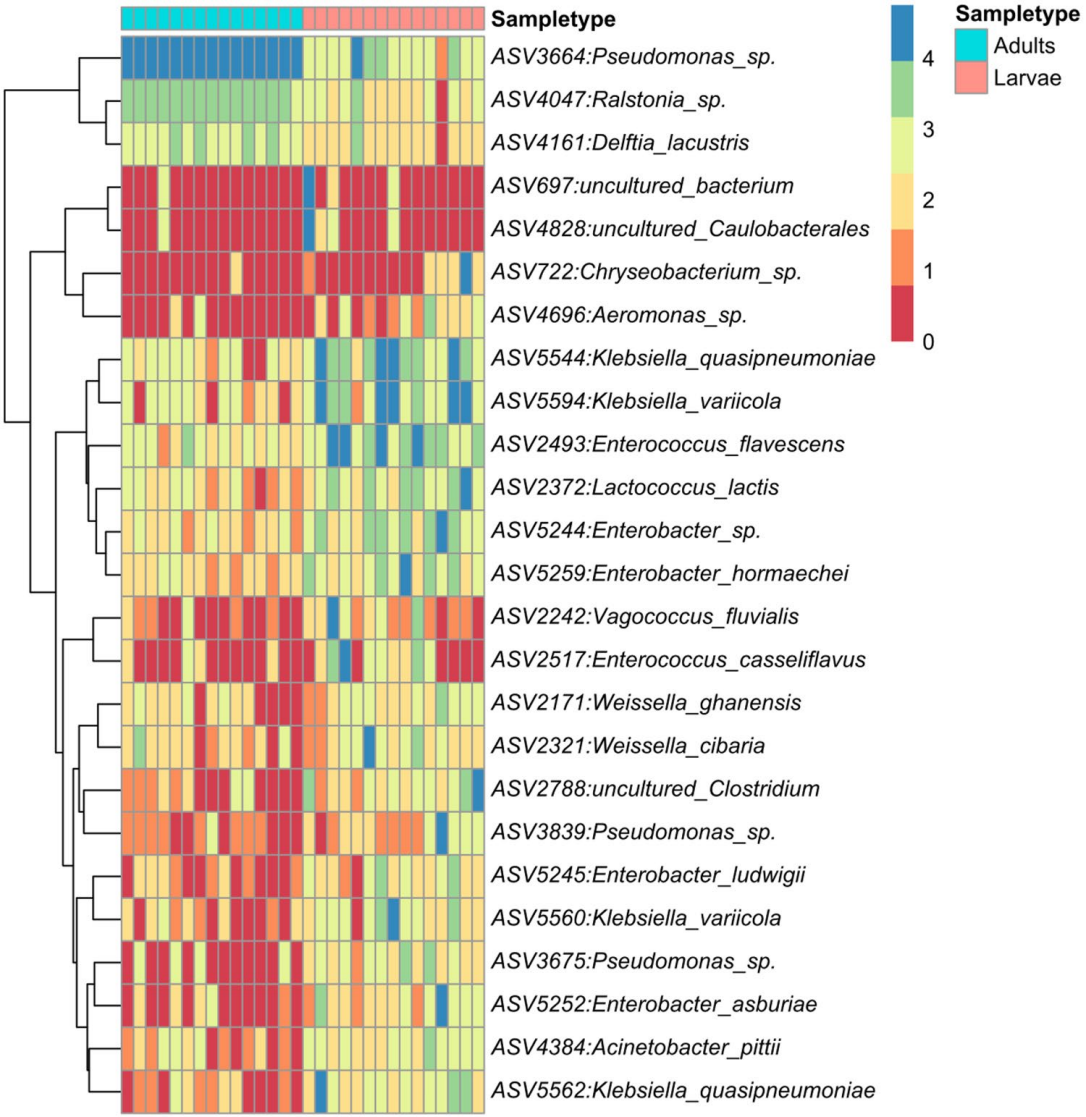
Top Abundant Bacterial species based on sample type ranging from 0 (least abundant) to 4 (most abundant).

Also at the Order taxonomic rank, Pseudomonadales were more abundant in the adult samples which gradually decreased in the larvae while Enterobacterales and Lactobacillales were more abundant in the larvae compared to the adults (Supplementary Fig. S2). A similar changing pattern of relative abundance in the adults and larvae were also obtained at the genus and species level (Fig. 3) where *Pseudomonas* was more abundant in the adults which reduced drastically in the larvae while *Klebsiella*, *Enterococcus* and *Enterobacter* had the opposite.

A total of 792 bacteria genera (Supplementary Table S2) were present in both the adult and larvae samples with the top 10 members of the group including *Pseudomonas, Enterococcus, Klebsiella, Enterobacter, Ralstonia, Erysipelatoclostridium, Lactococcus, Weissella, Aeromonas*, and *Stenotrophomonas* in decreasing other of relative abundance. A similar number of genera 264 and 139 were unique to the adults and larvae respectively while 389 genera were shared (Fig. 3). All the top 10 most abundant genera were not unique to either the adults or larvae but were found overlapping (shared) between the two sample types.

The genera *Aurantisolimonas, Luteolibacter, Filimonas, Edaphobaculum, Siphonobacter, Kerstersia* and *Peredibacter* were exclusively found in the larvae, *Eubacteriaceae, Muribaculaceae, Kerstersia* and *Romboutsia* were exclusively found in the adults.

**Figure 4:**
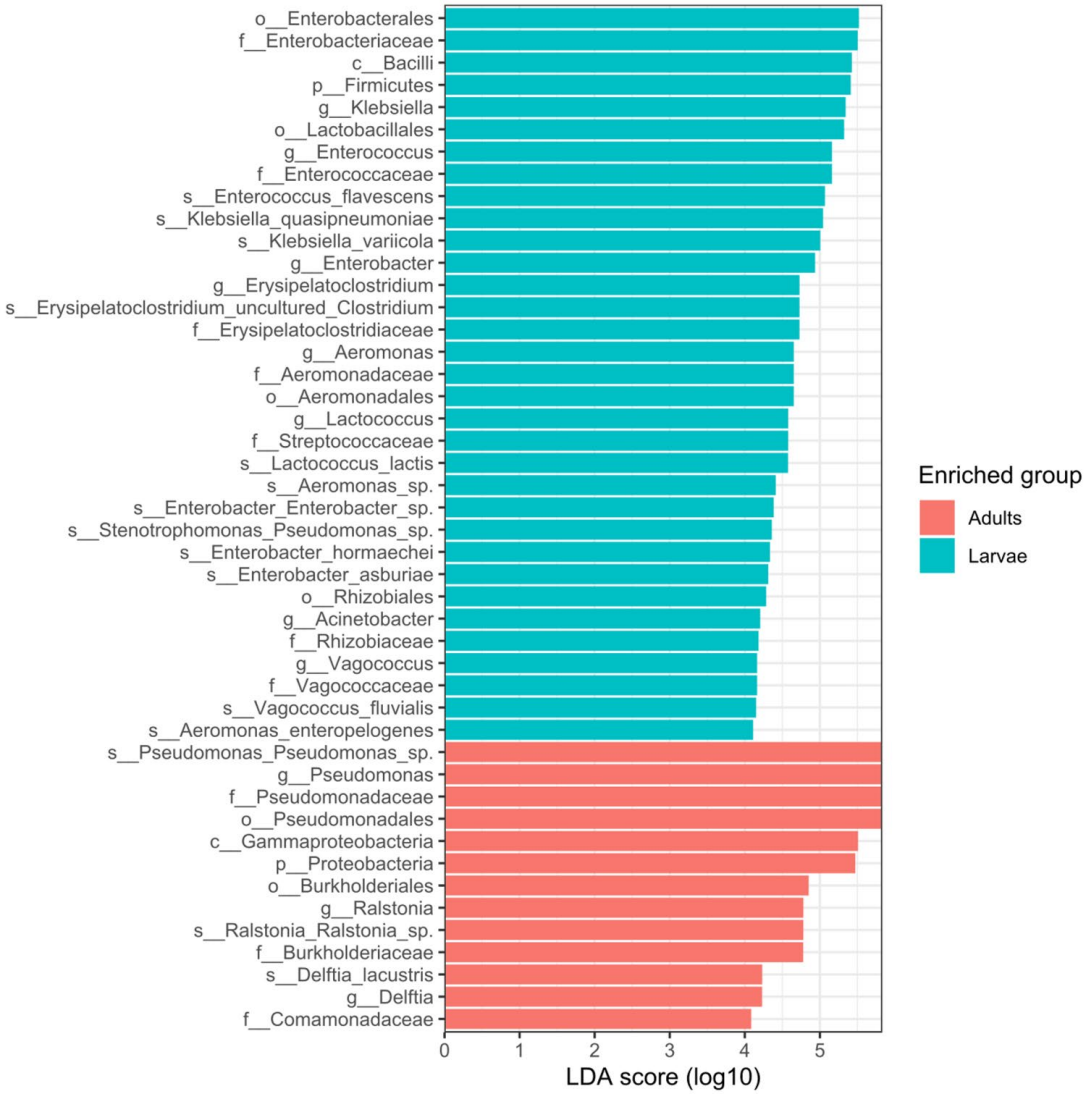
LEfSe result based on differentially abundant taxa in the larvae as compared to that of the adult.

**Figure 5:**
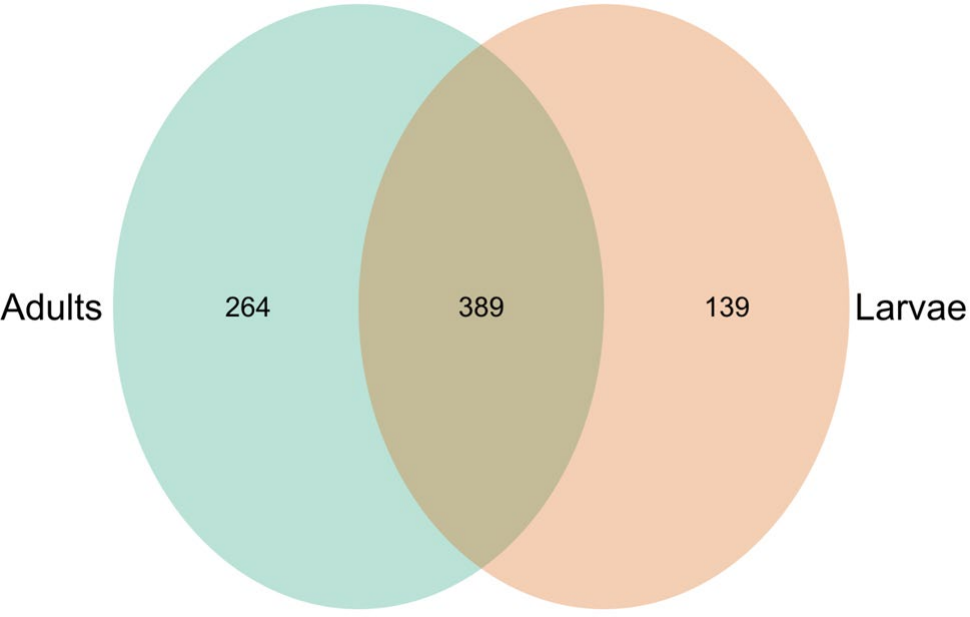
The Shared and Unique bacterial ASVs in each sample type.

Significant differences in the bacterial taxa were also identified by linear discriminant analysis effect size (LEfSe) comparison. The LEfSe chart (Fig. 4) showed that *Lactococcus*, *Enterococcus*, *Enterobacter* and *Klebsiella* were differentially abundant in the larvae while *Pseudomonas*, *Ralstonia*, *Delftia* were differentially abundant in the adults. This shows that the larvae samples had more taxa signatures than the adults. This is also supported by the Venn diagram above (Fig. 5).

### Bacteria community richness and diversity of *T. absoluta* microbiome

**Figure 6:**
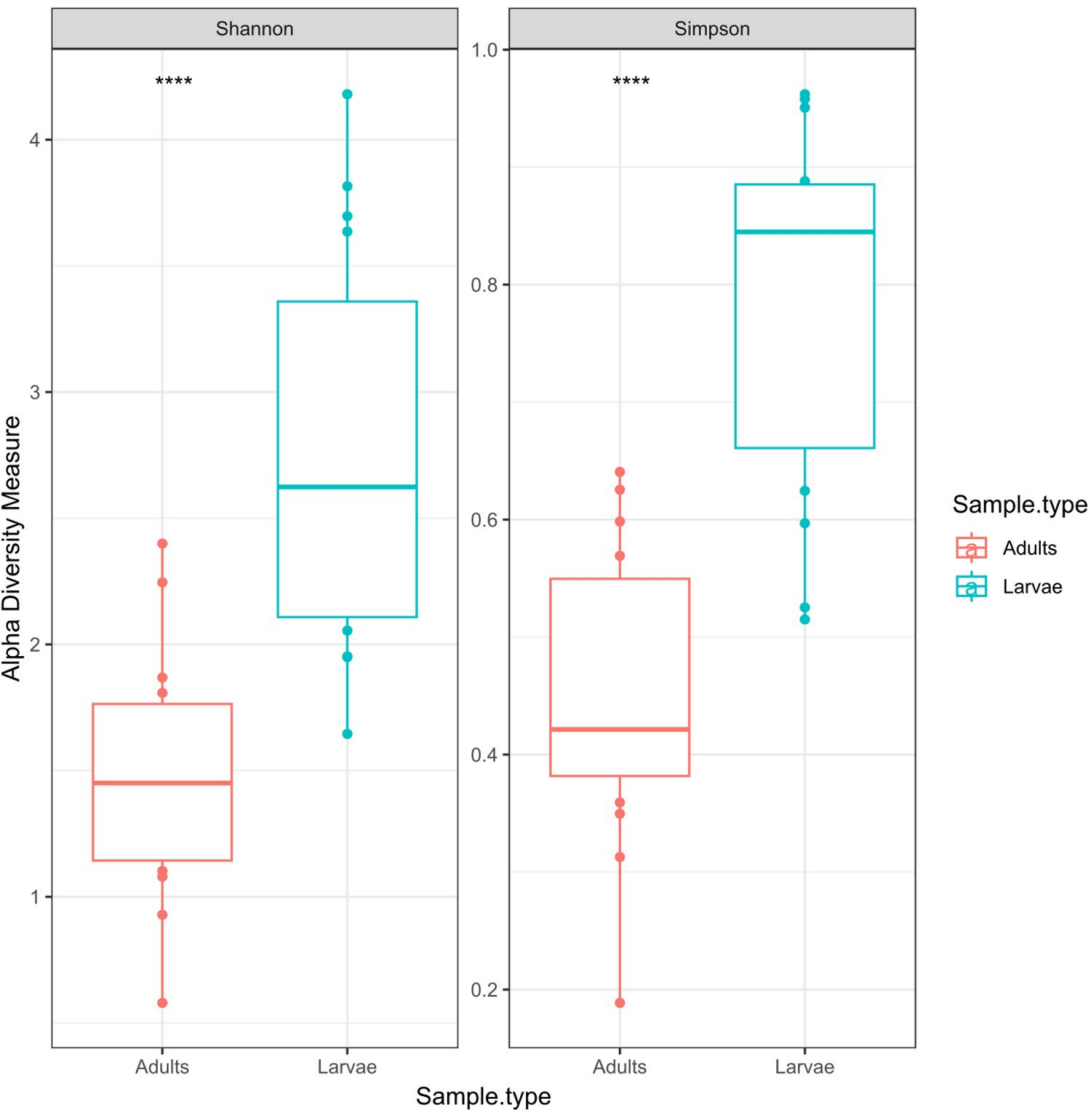
Bacterial communities Alpha diversity indices- Observed, Chao1, Shannon and Simpson of the adult and larva samples of *T. absoluta*.

There were differences between the larval (15 samples) and adults (15 samples) alpha diversities (Wilcoxon *P* ≤ 0.01) (Fig. 6). However, there were no significant differences in the alpha diversities (observed, Shannon and Simpson) between the larval samples from the 3 different (Kruskal–Wallis *P* > 0.05). Sample F1 from Southwest had the highest diversity species richness (chao1) with Shannon index of 4.04 (Supplementary Fig. S4) while sample K1 had the lowest diversity.

Within the adults, sample AH1 collected from Kazaure, Jigawa had the highest chao1 species richness index of 449 followed by sample AO1 from Rogo, Kano with 388 while sample AL1 from Rogo, Kano had the least richness (127.11) (Supplementary Fig. S5).

**Figure 7:**
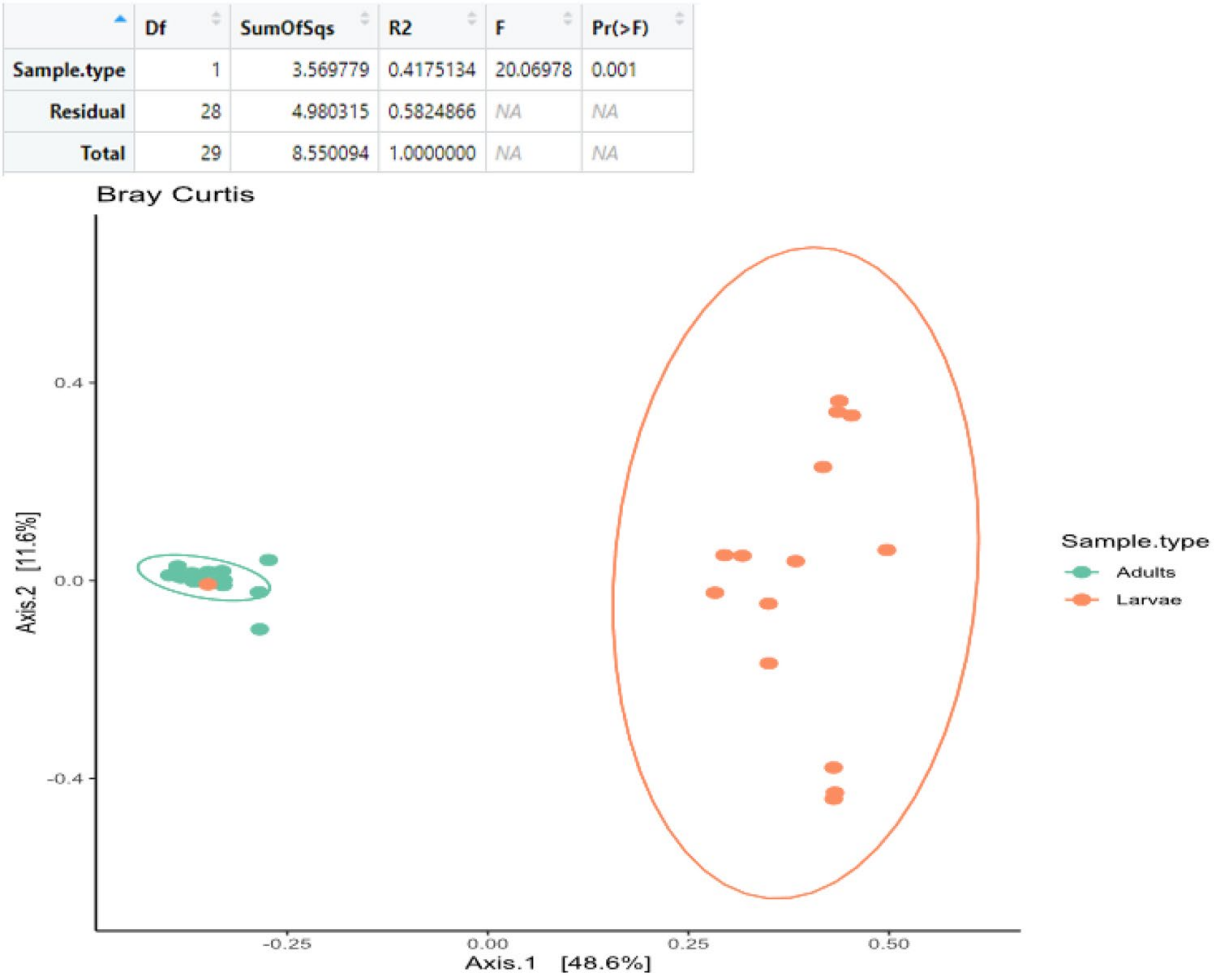
Principal co-ordinate analysis (PCoA) of the beta diversity of larval and adult samples beta diversity analyses by PERMANOVA at *P* < 0.01.

The principal coordinates analysis (PCoA) of the ASVs from the larval and adults samples showed distinct clustering which was confirmed significant by PERMANOVA at *P* < 0.01 (Fig. 7). Larval samples showed higher similarities with each other than with the adult samples and same pattern was observed with the adult-adult samples. Also, the samples did not differ based on location with the microbial communities having more similarities based on sample type.

### Microbial network interaction analysis of *T. absoluta* bacteria communities

**Figure 8:**
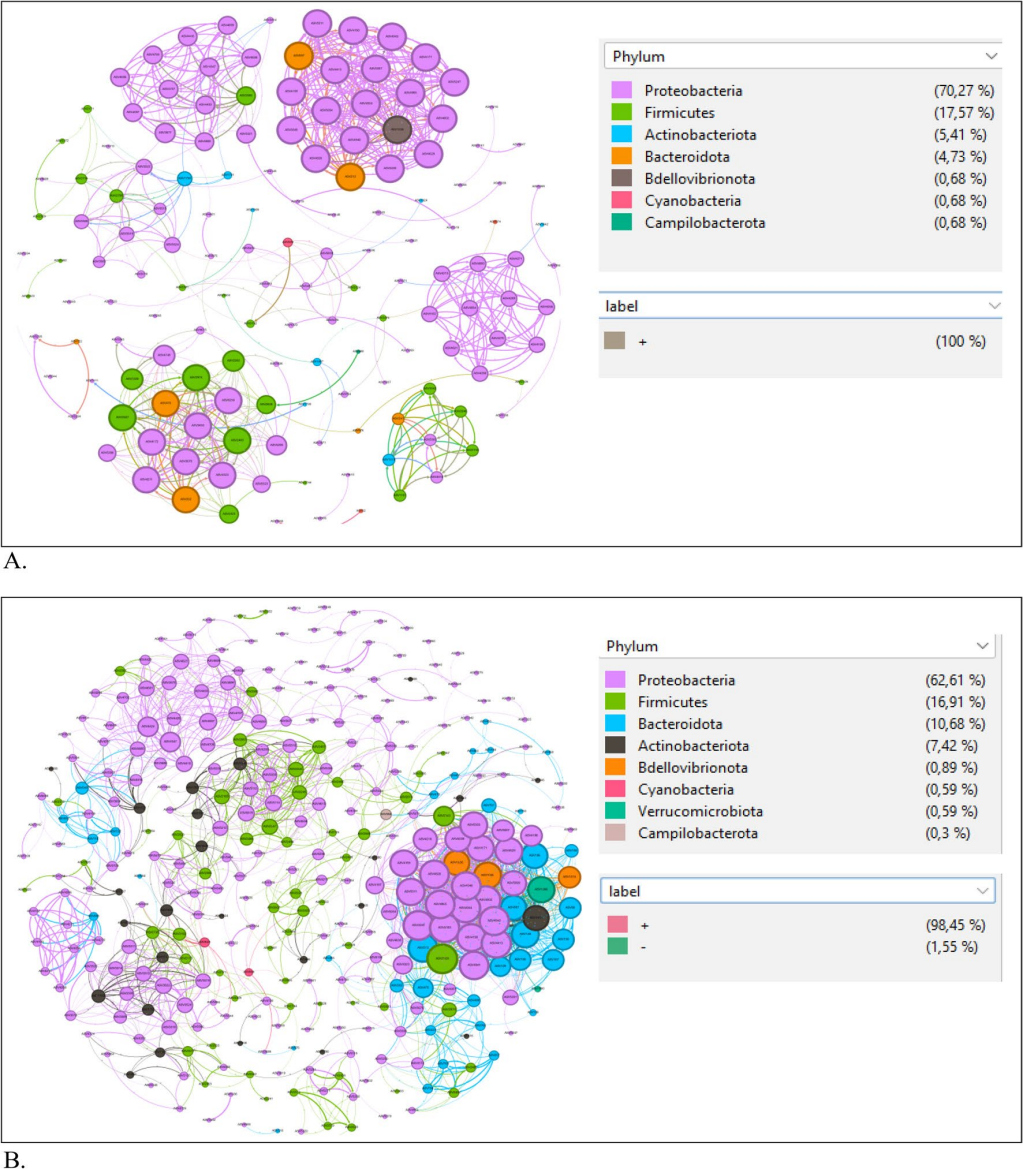
(**A**) Network analyses of the bacterial biota from adult samples. Each connection stands for a strong (Spearman's *ρ* > 0.7) and significant (*P*-value < 0.01) correlation. The size of each node is proportional to the number of its connections (degree). ASVs coloured by taxonomy. (**B**) Network analyses of the bacterial biota from larvae samples. Each connection stands for a strong (Spearman's *ρ* > 0.7) and significant (*P*-value < 0.01) correlation. The size of each node is proportional to the number of its connections (degree). ASVs coloured by taxonomy.

The inter-taxa correlations of the bacterial communities were revealed by the network analysis in Rstudio. The networks were explored and visualized with Gephi 0.10.1 (Bastian et al., 2009). The network topology indices such as modularity, clustering coefficient, average node connectivity, average path length and network diameter were calculated (Newman, 2003, 2006). The network analyses involved 150 and 337 core taxa in the adults (Fig. 8A) and larvae (Fig. 8B) respectively with Spearman correlation values ρ > 0.7, and *P* < 0.001. These core taxa had stronger and more significant connections between them in the larvae than in the adults. The phylum Proteobacteria had more interactions in both the larvae and adult bacterial communities. The average network distance between all pairs of taxa (average path length) and network diameter was lower in the adult bacterial communities (1.28 edges/diameter of 5) compared to (2.7 edges/diameter of 10) in the larvae respectively. The pattern of the network showed that the taxa tend to cluster together more in the adult than in the larvae with a clustering coefficient/modularity index of 0.29/0.79 for the adults and 0.27/0.72 for the larvae respectively.

Based on the weighted in-degree index from the different properties of the network analyses (Supplementary Tables S3 and S4), ASV5511 (*Bordetella genomosp*), ASV5247 (*Enterobacter kobei*), ASV5087 (*Rhizobium naphthalenivorans*), ASV5054 (*Kaistia* sp.) & ASV5039 (*Devosia* sp.) with ≥ 15 index were identified as the top keystone taxa in the adult bacterial communities while ASV5511 (*Bordetella genomosp*), ASV5503 (*Bordetella genomosp*), ASV4965 (*Sphingomonas* sp.), ASV4943 (*Sphingobium herbicidovorans*) and ASV 5099 (*Rhizobium* sp.) with ≥ 30 index were identified in the larvae.

### Potential functional analysis of *T. absoluta* bacteria microbiome

**Figure 9:**
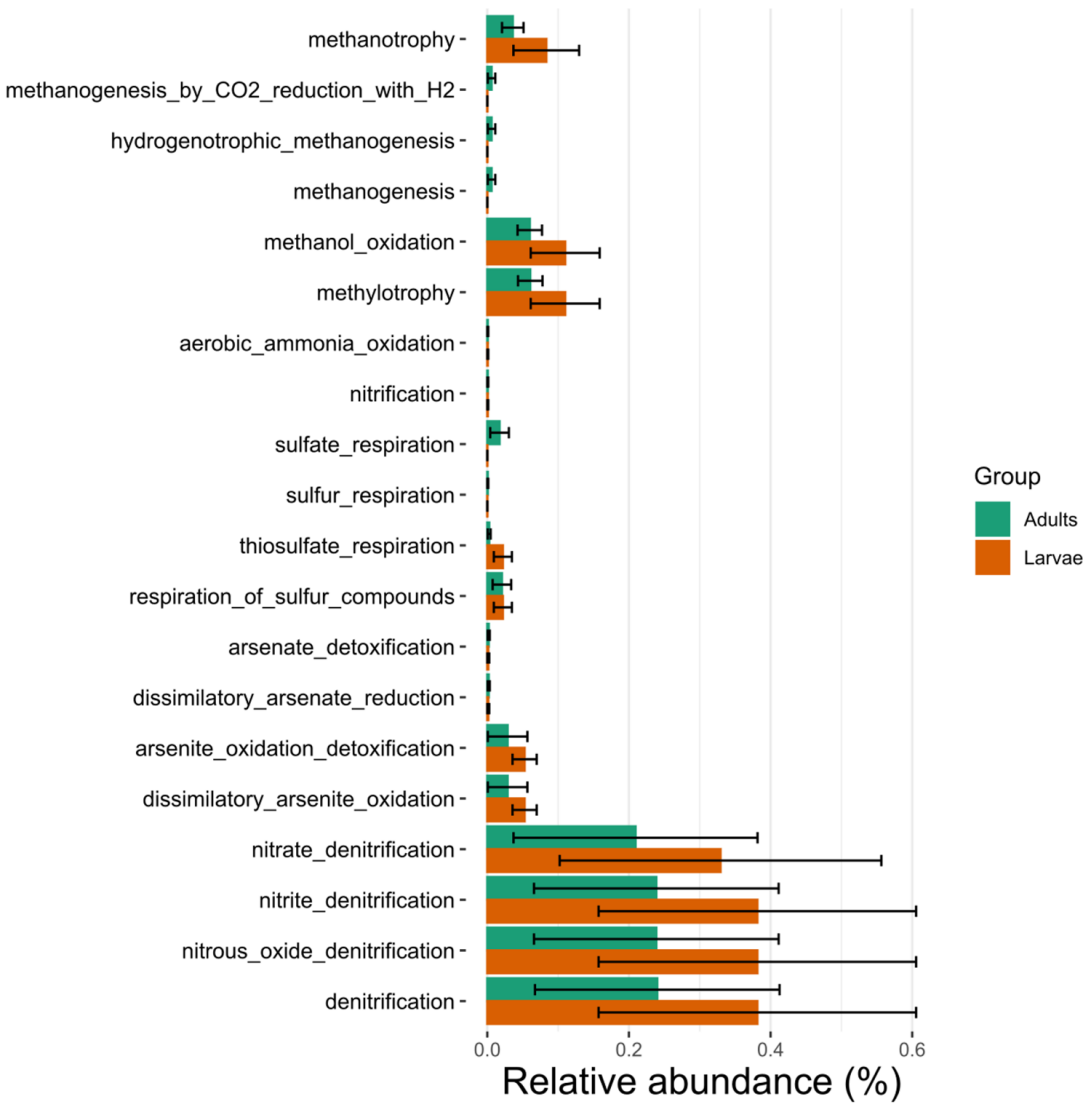
Abundance of the 20 predicted bacterial functions in the adult and larvae of *T. absoluta* as predicted by FAPROTAX.

Functional Annotation of Prokaryotic Taxa (FAPROTAX) was used to predict the functions of the bacterial communities of *T. absoluta* larvae and adults. A total of 20 putative functional groups (Fig. 9) were prominently identified such as sulphur respiration, arsenite reduction and oxidation as well as nitrification. Nitrous oxide denitrification and nitrite denitrification were the most dominant putative functions both in the adult and larvae bacterial biota followed by nitrate denitrification, dissimilatory arsenite oxidation, arsenite oxidation detoxification, methanol oxidation and methylotrophy.

There were no differences among the larvae and adults and bacterial functional groups (Fig. 9). However, the adults had an increased functional groups in sulfate respiration and methanogenesis activities.

## Discussion

In this study, the bacterial biota of *T. absoluta* were dominated by the phylum Proteobacteria with few Firmicutes which is similar to the only previous microbiome report on *T. absoluta* from China and Spain^27^. Proteobacteria has also been reported as dominant in other Lepidopteran insects such as in *Spodoptera littoralis*^52^, *Brithys crini*^53^ and *Cnaphalocrocis medinalis*^54^.

In contrast to the adults, the phylum Firmicutes followed by Proteobacteria were dominant in the larval samples of *T. absoluta* which is also in line with the larvae microbiome report of *Spodoptera littoralis*^52^, *Spodoptera frugiperda*^55–57^ and *Helicoverpa armigera*^58^.

Thakur et al.^52^ reported the dominance of the phylum Proteobacteria in adults of *Spodoptera littoralis* which was maintained in the eggs, reduced in the early-instar larvae, and then was gradually switched in the late-instar larvae where the Firmicutes were dominant with less Proteobacteria. This pattern possibly indicates that these bacterial biota are transmitted from adults to newborns via the eggs^52^. Proteobacteria which are mainly Gram negative are currently the largest phylum within the bacteria domain^59^ and Fimicutes which are mainly Gram positive and capable of breaking down carbohydrate in the human gut^60^. Our results are in agreement with previous reports that have shown that microbiomes of Lepidopteran insects are dominated by the phyla Proteobacteria and Firmicutes^61^.

The abundance of the genus *Pseudomonas* in the adult samples of *T. absoluta* has also been reported as dominant in Lepidopteran insects^61^ and it represents the core bacterial community in the adult samples together with *Ralstonia* and *Delftia*. The genus *Enterococcus* from the phylum Firmicutes which dominated the microbiome of the larval samples in this study has also been reported to be dominant in Lepidopteran insects^62,63^. *Klebsiella* and *Enterobacter* both from the phylum Proteobacteria were also reported present with relatively high abundance next to *Enterococcus* from the phylum Firmicutes from larvae of *Spodoptera frugiperda*^57^. *Enterococcus* were also found to be dominant in the microbiome study of larvae of *Spodoptera littoralis*^52^, *S. frugiperda*^56,57^ and from culture-dependent study of *S. frugiperda* larvae^57^. *Enterobacter* was also dominant in fresh larvae of mealworm larvae (*Tenebrio molitor*)^64^. Both *Enterobacter* and *Enterococcus* have been reported^65^ as dominant in adult *Drosophilia melanogaster*.

Bacterial genera such as *Weissella, Pseudomonas, Lactococcus, Ralstonia, Enterococcus, Delftia, Enterobacter, Rhizobium, Klebsiella and Achromobacter w*hich were observed in this study to be shared between the larvae and adult samples can persist as symbionts during the larval and adult stages^66–68^ with a considerable change in the composition from larvae to adults. This phenomenon is common in Lepidopteran insects, probably due to dramatic physiological and diet changes during metamorphosis^61^. It has been suggested that the crucial role of the microbiome in the development of insects is related to aspects such as immunity, reproduction, digestion, nutrition, and production of metabolites such as pheromones and antimicrobial molecules^69^. *Enterococcus* sp. isolated from the guts of *Plutella xylostella* enhance insecticide resistance to chlorpyrifos (organophosphate pesticide) by regulating the expression of an antimicrobial peptide named gloverin^63^. Insects may need specific bacteria such as *Enterococcus* sp. to degrade the toxic compounds like alkaloids and latex in their host plant as observed with the insects *Hyles euphorbiae* and *Brithys crini*^70^. *Enterococcus* spp. are also reported to be involved in plant defense suppression^71^.

The genus *Pseudomonas* is one of the most versatile bacterial genera with symbiotic relationship with their insect host^72^ and commonly found dominant in insects. It is believed to increase the genetic capacity of their hosts to degrade terpenes^73^ including plant defense suppression^71,74^ as well as providing pesticide resistance to their insect hosts^75^. Interestingly, *Pseudomonas* which was higher in the adults and disappeared in the larvae has been reported to have high larvicidal activity in *Spodoptera littura* larvae^18^. Several species of *Pseudomonas* such as *P. paralactis, Pseudomonas entomophila* and *Pseudomonas chlororaphis* have been reported to exhibit insecticidal activities against *Spodoptera littura*^17^, *Heliothis virescens* and *Plutella xylostella*^76^. In terms of diversity and richness, there were no significant difference both within the adult and larvae samples based on the different sample location which corroborates the reports of Wang et al.^27^ on *T. absoluta* from Spain, Xinjiang and Yunnan as well as that of Ugwu et al.^25^ on *Spodoptera frugiperda*. However, some reports revealed that environmental variability, developmental stage^52,77^ and diet^61,78^ can influence the insect microbiome. Furthermore, the community structures based on sample types (adults or larvae) were similar from the different agroecological locations.

The PCoA showed a significant difference in the beta diversity between the larval and adult samples which might be as a result of significant shifts in the microbiome as the larvae mature and metamorphosed into the adult stage^79^. It has been noted that insect developmental stages also have influence on the host microbiome as the composition, pattern, and relative abundance of each taxa changes^52,77,80^. The larvae had a core microbial taxa which were significantly different from those of the adults which was also supported by the LEfSe analyses. The biomarker discovery by LEfSe identifies the most biologically informative taxa distinguishing two or more genomic dataset^81^. In this study, the LEfSe result showed that the larvae samples have more taxa signatures than the adults. Gupta et al.^82^ found that differential abundant increase of *Pseudomonas* sp. improved pesticide resistance and survivability in adult brown planthopper (*Nilaparvata lugens*) causing damage in Rice fields. In the same way, Gong et al.^83^ showed that *Delftia* which was differentially abundant in *T. absoluta* adults has been linked with detoxification of insecticides and increased fitness in *Nilaparvata lugens*. These suggests that one of the ways which insects adapt to insecticides is by shifting its microbiome to interact with beneficial symbiont.

*Enterococcus* have also been reported to increase larval defense of *Bombyx mori* to biological control and removal of the *Enterococcus* symbiont from the larvae would increase the success of biocontrol of this insect larvae^84^. *Enterococcus* and *Lactococcus* can protect insect larvae against pathogens, plant toxins and increase host fitness^54,85^. *Enterococcus* is significantly abundant in *Hyles euphorbiae* and *Brithys crini*^70^ and other Lepidopteran larvae^86,87^.

The microbial network analyses explores the microbiome beyond the compositional and diversity inferences to reveal near real-world interactions between taxa. The co-occurrence pattern observed in this study indicate the presence of highly connected taxa of 4 nodes each on average. The clustering coefficient of the networks also indicate taxa from the same phylum clustered much more closely (77%) as expected in real-world microbial communities^88^. One of the most useful features of network analysis is that hubs (also termed keystone ASVs), which are taxa that are highly associated in a microbiome can be identified^89^. The network analyses clearly showed that ASV5511 (Bordetella genomosp.) identified as a keystone taxa in both adult and larvae bacterial microbiomes in *T. absoluta* had the most interactions with other taxa within the network. However, several other taxa also contributed to interactions within their hubs.

The predicted ecological functions of *T. absoluta* bacterial biota which showed a dominance of nitrous oxide and nitrite denitrification corroborates the results of the dominance of *Pseudomonas*, *Klebsiella*, *Enterobacter* and *Rhizobium* both in the network and taxonomic analyses. Denitrifying bacteria enhance the detoxification of organic insecticides in their host^63,90,91^. Furthermore, members of the Alicagenaceae, Pseudomonaceae and Rhizobiaceae to which most dominant and keystone taxa in this study belong to have been reported to have the As(III) oxidase gene^92,93^ which is responsible for oxidizing the arsenic found in some arsenic contaminated farms^93^. This result corroborates the recent study of Wang et al.^94^ on the abundance of nitrate reduction functions in bacterial communities of the brown planthopper (*Nilaparvata lugens)* destroying rice fields in Asia.

## Conclusion

With the continuous expansion in the geographic distribution of *T. absoluta*, characterizing the microbial diversity of *T. absoluta* is a significant step to developing alternative non-chemical methods to combat this pest*.* This study reveals the bacterial microbiomes of *T. absoluta*, their abundance, distribution, and interaction. Although no obvious impact on sampling sites was observed, a more detailed study involving samples from the six agroecological zones merit further investigation to ascertain any potential impact of geographical location on the microbiome. The results, however, could further contribute to the development and exploitation of the microbial symbionts and natural enemies of *T. absoluta* as an alternative to chemical control management strategy of this destructive invasive pest.

### Supplementary Information

Supplementary Information.

## Data Availability

The sequencing dataset can be found in online SRA repository of the National Center for Biotechnology Information (NCBI) under the BioProject ID PRJNA1032433.

## References

[1] Chakraborty S, Newton AC (2011). Climate change, plant diseases and food security: An overview. Plant Pathol..

[2] Qadri, M., Short, S., Gast, K., Hernandez, J. & Wong, A. C.-N. Microbiome innovation in agriculture: Development of microbial based tools for insect pest management. *Front. Sustain. Food Syst.* **4**, (2020).

[3] Tarusikirwa VL, Machekano H, Mutamiswa R, Chidawanyika F, Nyamukondiwa C (2020). Tuta absoluta (Meyrick) (Lepidoptera: Gelechiidae) on the “Offensive” in Africa: Prospects for integrated management initiatives. Insects.

[4] Sultan, A. M. S. A., Morsi, G. A. M., El-Fayoumi, H. M. & Abdel-Baki, A.-A. S. Population Dynamics of the Leaf Miner Tuta absoluta (Lepidoptra: Gelechiidae) and its Parasitoids on Tomato Crop in Beni-Suef, Egypt. *Adv. Anim. Vet. Sci.* **10**, (2022).

[5] Desneux N, Luna MG, Guillemaud T, Urbaneja A (2011). The invasive South American tomato pinworm, Tuta absoluta, continues to spread in Afro-Eurasia and beyond: The new threat to tomato world production. J. Pest Sci..

[6] Desneux N (2010). Biological invasion of European tomato crops by Tuta absoluta: Ecology, geographic expansion and prospects for biological control. J. Pest Sci..

[7] De Smedt C, Van Damme V, De Clercq P, Spanoghe P (2016). Insecticide effect of zeolites on the Tomato Leafminer Tuta absoluta (Lepidoptera: Gelechiidae). Insects.

[8] Roditakis E (2015). First report of Tuta absoluta resistance to diamide insecticides. J. Pest Sci..

[9] Haddi K (2017). Mutation in the ace-1 gene of the tomato leaf miner (Tuta absoluta) associated with organophosphates resistance. J. Appl. Entomol..

[10] Zibaee I, Mahmood K, Esmaeily M, Bandani AR, Kristensen M (2018). Organophosphate and pyrethroid resistances in the tomato leaf miner Tuta absoluta (Lepidoptera: Gelechiidae) from Iran. J. Appl. Entomol..

[11] Silva TBM (2016). Susceptibility levels of Tuta absoluta (Meyrick) (Lepidoptera: Gelechiidae) to minor classes of insecticides in Brazil. Crop Prot..

[12] Guedes RNC, Siqueira HAA (2012). The tomato borer Tuta absoluta: Insecticide resistance and control failure. CABI Rev..

[13] Truman JW, Riddiford LM (2019). The evolution of insect metamorphosis: A developmental and endocrine view. Philos. Trans. R. Soc. Lond. B. Biol. Sci..

[14] Pandey M (2023). A review on biology and possible management strategies of tomato leaf miner, Tuta absoluta (Meyrick), Lepidoptera: Gelechiidae in Nepal. Heliyon.

[15] van den Bosch TJM, Welte CU (2016). Detoxifying symbionts in agriculturally important pest insects. Microb. Biotechnol..

[16] Dhanapal R, Kumar DVSR, Lakshmipathy R, Rani CS, Kumar VM (2020). Exploration of indigenous strains of the green muscardine fungus from soils and their pathogenicity against the tobacco caterpillar, Spodoptera litura (Fabricius) (Lepidoptera: Noctuidae). Egypt. J. Biol. Pest Control.

[17] Devi, S., Saini, H. S. & Kaur, S. Insecticidal and growth inhibitory activity of gut microbes isolated from adults of Spodoptera litura (Fab.). *BMC Microbiol.* **22**, 71 (2022). 10.1186/s12866-022-02476-3 PMC8908599 35272633

[18] Sarkhandia, S. *et al.* Larvicidal, growth inhibitory and biochemical effects of soil bacterium, Pseudomonas sp. EN4 against Spodoptera litura (Fab.) (Lepidoptera: Noctuidae). *BMC Microbiol.* **23**, 95 (2023). 10.1186/s12866-023-02841-w PMC10069027 37013477

[19] Ziganshina EE (2018). Fungal, bacterial, and archaeal diversity in the digestive tract of several beetle larvae (Coleoptera). BioMed. Res. Int..

[20] Lv D (2021). Comparison of gut bacterial communities of fall armyworm (Spodoptera frugiperda) reared on different host plants. int. J. Mol. Sci..

[21] Gurung, K., Wertheim, B. & Falcao Salles, J. The microbiome of pest insects: It is not just bacteria. *Entomol. Exp. Appl.* **167**, 156–170 (2019).

[22] Yao Z (2019). Similar shift patterns in gut bacterial and fungal communities across the life stages of Bactrocera minax Larvae from two field populations. Front. Microbiol..

[23] Lu M, Hulcr J, Sun J (2016). The role of symbiotic microbes in insect invasions. Annu. Rev. Ecol. Evol. Syst..

[24] Crotti E (2012). Microbial symbionts: A resource for the management of insect-related problems. Microb. Biotechnol..

[25] Ugwu, J. A., Liu, M., Sun, H. & Asiegbu, F. O. Microbiome of the larvae of Spodoptera frugiperda (JE Smith) (Lepidoptera: Noctuidae) from maize plants. *J. Appl. Entomol.* 764–776 (2020).

[26] Chen J (2023). The dynamics of the microbial community in fall armyworm Spodoptera frugiperda during a life cycle. Entomol. Exp. Appl..

[27] Wang H (2022). Similar bacterial communities among different populations of a newly emerging invasive species, Tuta absoluta (Meyrick). Insects.

[28] Salako FK, Tian G, Kirchhof G, Akinbola GE (2006). Soil particles in agricultural landscapes of a derived savanna in southwestern Nigeria and implications for selected soil properties. Geoderma.

[29] Oladoye AO, Aduradola AM, Bada BS, Kudaisi BO (2011). Light fraction of soil organic matter under different management systems in Abeokuta, a derived Savanna. Nigeria. J. Agric. For. Soc. Sci..

[30] Adekola OF, Affinnih KO, Musa AK, Oluleye F, Ajayi CO (2014). Evaluation of growth, yield and susceptibility of Five Cassava Cultivars in Ilorin, Southern Guinea Savanna. Nigeria. Ilorin J. Sci..

[31] Yamusa, A. M. & Abdulkadir (Mrs.), A. Rainfall and Temperature trends in Samaru and Minjibir, Northern Guinea and Sudan Savannas of Nigeria. *J. Sustain. Agric. Environ. JSAE* **18**, 255–265 (2020).

[32] Asiegbu FO, Abu S, Stenlid J, Johansson M (2004). Sequence polymorphism and molecular characterization of laccase genes of the conifer pathogen Heterobasidion annosum. Mycol. Res..

[33] Klindworth A (2013). Evaluation of general 16S ribosomal RNA gene PCR primers for classical and next-generation sequencing-based diversity studies. Nucleic Acids Res..

[34] Cock PJA, Fields CJ, Goto N, Heuer ML, Rice PM (2010). The Sanger FASTQ file format for sequences with quality scores, and the Solexa/Illumina FASTQ variants. Nucleic Acids Res..

[35] Magoč T, Salzberg SL (2011). FLASH: Fast length adjustment of short reads to improve genome assemblies. Bioinformatics.

[36] Caporaso JG (2010). QIIME allows analysis of high-throughput community sequencing data. Nat. Methods.

[37] Callahan BJ (2016). DADA2: High-resolution sample inference from Illumina amplicon data. Nat. Methods.

[38] Robeson MS (2021). RESCRIPt: Reproducible sequence taxonomy reference database management. PLoS Comput. Biol..

[39] Quast C (2013). The SILVA ribosomal RNA gene database project: Improved data processing and web-based tools. Nucleic Acids Res..

[40] Chao A, Lee S-M (1992). Estimating the number of classes via sample coverage. J. Am. Stat. Assoc..

[41] Chao A (1984). nonparametric estimation of the number of classes in a population. Scand. J. Stat..

[42] Kim B-R (2017). Deciphering diversity indices for a better understanding of microbial communities..

[43] Bolyen E (2019). Reproducible, interactive, scalable and extensible microbiome data science using QIIME 2. Nat. Biotechnol..

[44] Wickham, H. *ggplot2: Elegant Graphics for Data Analysis*. (Springer, 2009). 10.1007/978-0-387-98141-3.

[45] RStudio Team. RStudio: Integrated Development for R. RStudio, PBC, Boston, MA URL . *Posit Support* http://www.rstudio.com/ (2023).

[46] R Core Team. R: A language and environment for statistical computing. R Foundation for Statistical Computing, Vienna, Austria. https://www.r-project.org/ (2023).

[47] McMurdie PJ, Holmes S (2013). phyloseq: An R package for reproducible interactive analysis and graphics of microbiome census data. PLOS ONE.

[48] Wickham H (2019). Welcome to the Tidyverse. J. Open Source Softw..

[49] de Vries FT (2018). Soil bacterial networks are less stable under drought than fungal networks. Nat. Commun..

[50] Louca S, Doebeli M, Parfrey LW (2018). Correcting for 16S rRNA gene copy numbers in microbiome surveys remains an unsolved problem. Microbiome.

[51] Sansupa, C., Fareed Mohamed Wahdan, S., Disayathanoowat, T. & Purahong, W. Identifying hidden viable bacterial taxa in tropical forest soils using amplicon sequencing of enrichment cultures. *Biology* **10**, 569 (2021). 10.3390/biology10070569 PMC8301126 34206701

[52] Chen B (2016). Biodiversity and activity of the gut microbiota across the life history of the insect herbivore Spodoptera littoralis. Sci. Rep..

[53] González-Serrano F (2020). The Gut Microbiota composition of the Moth Brithys crini Reflects Insect Metamorphosis. Microb. Ecol..

[54] Yang Y, Liu X, Xu H, Liu Y, Lu Z (2022). Effects of Host Plant and Insect Generation on Shaping of the Gut Microbiota in the Rice Leaffolder. Cnaphalocrocis medinalis. Front. Microbiol..

[55] Chen Y (2021). Gut Microbiota Dysbiosis Influences Metabolic Homeostasis in Spodoptera frugiperda. Front. Microbiol..

[56] Gomes AFF, Omoto C, Cônsoli FL (2020). Gut bacteria of field-collected larvae of Spodoptera frugiperda undergo selection and are more diverse and active in metabolizing multiple insecticides than laboratory-selected resistant strains. J. Pest Sci..

[57] Higuita Palacio, M. F. *et al.* Dry and Rainy Seasons Significantly Alter the Gut Microbiome Composition and Reveal a Key Enterococcus sp. (Lactobacillales: Enterococcaceae) Core Component in Spodoptera frugiperda (Lepidoptera: Noctuidae) Corn strain From Northwestern Colombia. *J. Insect Sci. Online* **21**, 10 (2021). 10.1093/jisesa/ieab076 PMC8567080 34734290

[58] Priya NG, Ojha A, Kajla MK, Raj A, Rajagopal R (2012). Host Plant Induced Variation in Gut Bacteria of Helicoverpa armigera. PLOS ONE.

[59] Rizzatti G, Lopetuso LR, Gibiino G, Binda C, Gasbarrini A (2017). Proteobacteria: A common factor in human diseases. BioMed Res. Int..

[60] Ottman N, Smidt H, de Vos WM, Belzer C (2012). The function of our microbiota: Who is out there and what do they do?. Front. Cell. Infect. Microbiol..

[61] Paniagua Voirol, L. R., Frago, E., Kaltenpoth, M., Hilker, M. & Fatouros, N. E. Bacterial Symbionts in Lepidoptera: Their Diversity, Transmission, and Impact on the Host. *Front. Microbiol.* **9**, (2018). 10.3389/fmicb.2018.00556 PMC5881003 29636736

[62] Thakur A, Dhammi P, Saini HS, Kaur S (2015). Pathogenicity of bacteria isolated from gut of Spodoptera litura (Lepidoptera: Noctuidae) and fitness costs of insect associated with consumption of bacteria. J. Invertebr. Pathol..

[63] Xia, X. *et al.* Gut Microbiota Mediate Insecticide Resistance in the Diamondback Moth, Plutella xylostella (L.). *Front. Microbiol.* **9**, (2018). 10.3389/fmicb.2018.00025 PMC5787075 29410659

[64] Wynants E (2017). Effect of post-harvest starvation and rinsing on the microbial numbers and the bacterial community composition of mealworm larvae (Tenebrio molitor). Innov. Food Sci. Emerg. Technol..

[65] Cox CR, Gilmore MS (2007). Native microbial colonization of Drosophila melanogaster and its use as a model of Enterococcus faecalis pathogenesis. Infect. Immun..

[66] Vasanthakumar A, Handelsman J, Schloss PD, Bauer LS, Raffa KF (2008). Gut Microbiota of an Invasive Subcortical Beetle, Agrilus planipennis Fairmaire across various life stages. Environ. Entomol..

[67] Lauzon CR, Mccombs SD, Potter SE, Peabody NC (2009). Establishment and vertical passage of Enterobacter (Pantoea) Agglomerans and Klebsiella pneumoniae through all life stages of the mediterranean Fruit Fly (Diptera: Tephritidae). Ann. Entomol. Soc. Am..

[68] Arias-Cordero E (2012). Comparative evaluation of the gut microbiota associated with the below- and above-ground life stages (larvae and beetles) of the forest cockchafer, Melolontha hippocastani. PloS One.

[69] Dillon RJ, Dillon VM (2004). The gut bacteria of insects: nonpathogenic interactions. Annu. Rev. Entomol..

[70] Vilanova C, Baixeras J, Latorre A, Porcar M (2016). The generalist inside the specialist: Gut bacterial communities of two insect species feeding on toxic plants are dominated by Enterococcus sp. Front. Microbiol..

[71] Chung SH (2013). Herbivore exploits orally secreted bacteria to suppress plant defenses. Proc. Natl. Acad. Sci..

[72] Teoh, M.-C., Furusawa, G. & Veera Singham, G. Multifaceted interactions between the pseudomonads and insects: mechanisms and prospects. *Arch. Microbiol.* **203**, 1891–1915 (2021). 10.1007/s00203-021-02230-9 33634321

[73] Adams AS (2013). Mountain pine beetles colonizing historical and naive host trees are associated with a bacterial community highly enriched in genes contributing to terpene metabolism. Appl. Environ. Microbiol..

[74] Consales F (2012). Insect oral secretions suppress wound-induced responses in Arabidopsis. J. Exp. Bot..

[75] de Almeida LG, de Moraes LAB, Trigo JR, Omoto C, Cônsoli FL (2017). The gut microbiota of insecticide-resistant insects houses insecticide-degrading bacteria: A potential source for biotechnological exploitation. PLoS ONE.

[76] Ruffner B (2013). Oral insecticidal activity of plant-associated pseudomonads. Environ. Microbiol..

[77] Gao X (2019). Biodiversity of the microbiota in Spodoptera exigua (Lepidoptera: Noctuidae). J. Appl. Microbiol..

[78] Zheng Y (2020). Midgut microbiota diversity of potato tuber moth associated with potato tissue consumed. BMC Microbiol..

[79] Dee Tan IY, Bautista MAM (2022). Bacterial survey in the guts of domestic silkworms, Bombyx mori L.. Insects.

[80] Yun J-H (2014). Insect gut bacterial diversity determined by environmental habitat, diet, developmental stage, and phylogeny of host. Appl. Environ. Microbiol..

[81] Segata N (2011). Metagenomic biomarker discovery and explanation. Genome Biol..

[82] Gupta, A., Sinha, D. K. & Nair, S. Shifts in Pseudomonas species diversity influence adaptation of brown planthopper to changing climates and geographical locations. *iScience* **25**, 104550 (2022). 10.1016/j.isci.2022.104550 PMC9218508 35754716

[83] Gong G (2023). Nicotine perturbs the microbiota of brown planthopper (Nilaparvata lugens stål Hemiptera: Delphinidae). Ecotoxicol. Environ. Saf..

[84] Zhang X (2022). The gut commensal bacterium Enterococcus faecalis LX10 contributes to defending against Nosema bombycis infection in Bombyx mori. Pest. Manag. Sci..

[85] Shao Y (2017). Symbiont-derived antimicrobials contribute to the control of the lepidopteran gut microbiota. Cell Chem. Biol..

[86] Liu Y (2020). Comparison of gut bacterial communities and their associations with host diets in four fruit borers. Pest. Manag. Sci..

[87] Wang, X. *et al.* Variability of Gut Microbiota Across the Life Cycle of Grapholita molesta (Lepidoptera: Tortricidae). *Front. Microbiol.* **11**, (2020). 10.3389/fmicb.2020.01366 PMC7340173 32714300

[88] Barberán A, Bates ST, Casamayor EO, Fierer N (2012). Using network analysis to explore co-occurrence patterns in soil microbial communities. ISME J..

[89] Banerjee S (2021). Microbial interkingdom associations across soil depths reveal network connectivity and keystone taxa linked to soil fine-fraction carbon content. Agric. Ecosyst. Environ..

[90] Ramya, S. L., Venkatesan, T., Srinivasa Murthy, K., Jalali, S. K. & Verghese, A. Detection of carboxylesterase and esterase activity in culturable gut bacterial flora isolated from diamondback moth, Plutella xylostella (Linnaeus), from India and its possible role in indoxacarb degradation. *Braz. J. Microbiol. Publ. Braz. Soc. Microbiol.* **47**, 327–336 (2016). 10.1016/j.bjm.2016.01.012 PMC4874610 26991291

[91] Tago K, Kikuchi Y, Nakaoka S, Katsuyama C, Hayatsu M (2015). Insecticide applications to soil contribute to the development of Burkholderia mediating insecticide resistance in stinkbugs. Mol. Ecol..

[92] Warelow TP, Pushie MJ, Cotelesage JJH, Santini JM, George GN (2017). The active site structure and catalytic mechanism of arsenite oxidase. Sci. Rep..

[93] Upadhyay MK, Shukla A, Yadav P, Srivastava S (2019). A review of arsenic in crops, vegetables, animals and food products. Food Chem..

[94] Wang Z-L, Pan H, Wu W, Li M-Y, Yu X-P (2021). The gut bacterial flora associated with brown planthopper is affected by host rice varieties. Arch. Microbiol..

