# Supplementary material for: Bacterial biota associated with the invasive insect pest Tuta absoluta (Meyrick)
Source: Sci Rep. 2024 Apr 9;14:8268. doi: 10.1038/s41598-024-58753-w (PMC11003966; doi:10.1038/s41598-024-58753-w)
Supplement: Supplementary file 1 — Supplementary Information. [file 41598_2024_58753_MOESM1_ESM.docx]

**SUPPLEMENTARY FILES**

Supplementary Table 1. Results of quality filtering of the Sequences with Dada2

| Sample ID | input | filtered | % passed filter | denoised | merged | % merged | non-chimeric | % non-chimeric |
| --- | --- | --- | --- | --- | --- | --- | --- | --- |
| AA1 | 76604 | 68294 | 89.15 | 67677 | 66530 | 86.85 | 64065 | 83.63 |
| AB1 | 85146 | 77315 | 90.8 | 76356 | 73926 | 86.82 | 68709 | 80.7 |
| AC1 | 39821 | 35413 | 88.93 | 34564 | 32958 | 82.77 | 31101 | 78.1 |
| AD1 | 69672 | 63231 | 90.76 | 62338 | 60142 | 86.32 | 56080 | 80.49 |
| AE1 | 82371 | 73146 | 88.8 | 72448 | 70824 | 85.98 | 66649 | 80.91 |
| AF1 | 60599 | 54178 | 89.4 | 53431 | 51636 | 85.21 | 47961 | 79.14 |
| AG1 | 84401 | 77466 | 91.78 | 76708 | 74993 | 88.85 | 69327 | 82.14 |
| AH1 | 84822 | 66250 | 78.1 | 64090 | 60636 | 71.49 | 57575 | 67.88 |
| AI1 | 75290 | 69284 | 92.02 | 68632 | 67286 | 89.37 | 63415 | 84.23 |
| AJ1 | 65807 | 60075 | 91.29 | 59188 | 57140 | 86.83 | 53081 | 80.66 |
| AK1 | 54699 | 50321 | 92 | 49476 | 47734 | 87.27 | 44356 | 81.09 |
| AL1 | 56713 | 51581 | 90.95 | 51232 | 50721 | 89.43 | 49435 | 87.17 |
| AM1 | 55545 | 50953 | 91.73 | 50584 | 49879 | 89.8 | 47162 | 84.91 |
| AN1 | 62806 | 57646 | 91.78 | 56949 | 55623 | 88.56 | 53114 | 84.57 |
| AO1 | 60685 | 55146 | 90.87 | 53997 | 52215 | 86.04 | 51175 | 84.33 |
| F1 | 102683 | 96211 | 93.7 | 95454 | 92940 | 90.51 | 88861 | 86.54 |
| G1 | 116205 | 109343 | 94.09 | 108717 | 106616 | 91.75 | 102534 | 88.24 |
| H1 | 113457 | 107505 | 94.75 | 106862 | 104852 | 92.42 | 90961 | 80.17 |
| J1 | 104454 | 98083 | 93.9 | 97098 | 94861 | 90.82 | 90213 | 86.37 |
| K1 | 47243 | 42199 | 89.32 | 41616 | 40370 | 85.45 | 37637 | 79.67 |
| Q1 | 102005 | 96542 | 94.64 | 95705 | 93128 | 91.3 | 86180 | 84.49 |
| R1 | 107782 | 101170 | 93.87 | 100420 | 98745 | 91.62 | 92432 | 85.76 |
| S1 | 114696 | 108751 | 94.82 | 108147 | 106005 | 92.42 | 102707 | 89.55 |
| T1 | 110519 | 102983 | 93.18 | 102046 | 99293 | 89.84 | 73107 | 66.15 |
| U1 | 101178 | 95347 | 94.24 | 94614 | 92256 | 91.18 | 80485 | 79.55 |
| V1 | 103058 | 96637 | 93.77 | 95579 | 92832 | 90.08 | 78666 | 76.33 |
| W1 | 109728 | 103344 | 94.18 | 102343 | 99589 | 90.76 | 88054 | 80.25 |
| X1 | 117489 | 110164 | 93.77 | 109425 | 107273 | 91.3 | 98080 | 83.48 |
| Y1 | 102884 | 95709 | 93.03 | 94983 | 92422 | 89.83 | 78086 | 75.9 |
| Z1 | 93591 | 87854 | 93.87 | 86731 | 84085 | 89.84 | 80807 | 86.34 |


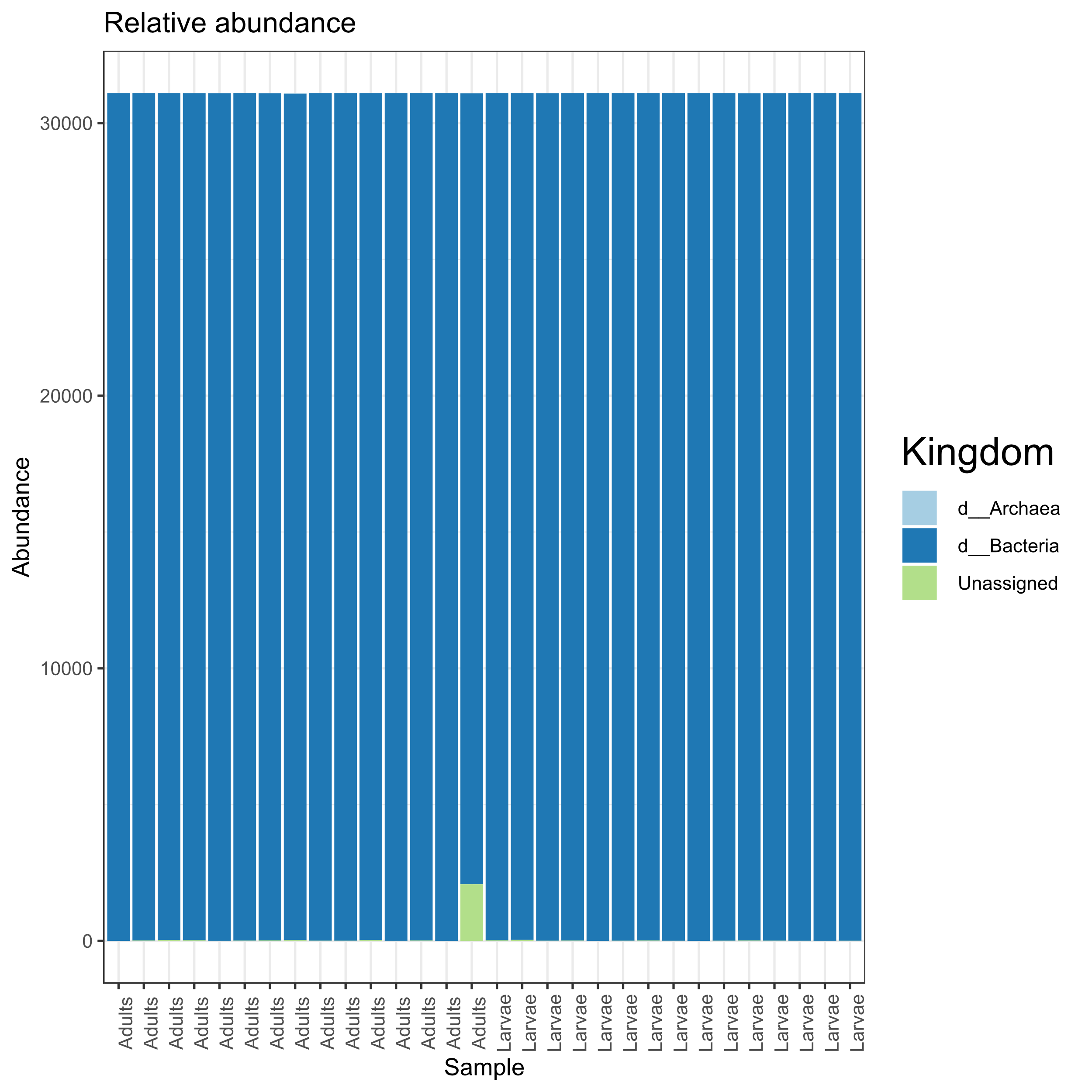
Supplementary Figure 1. Taxa Bar showing all the ASVs that belong to bacteria per sample.


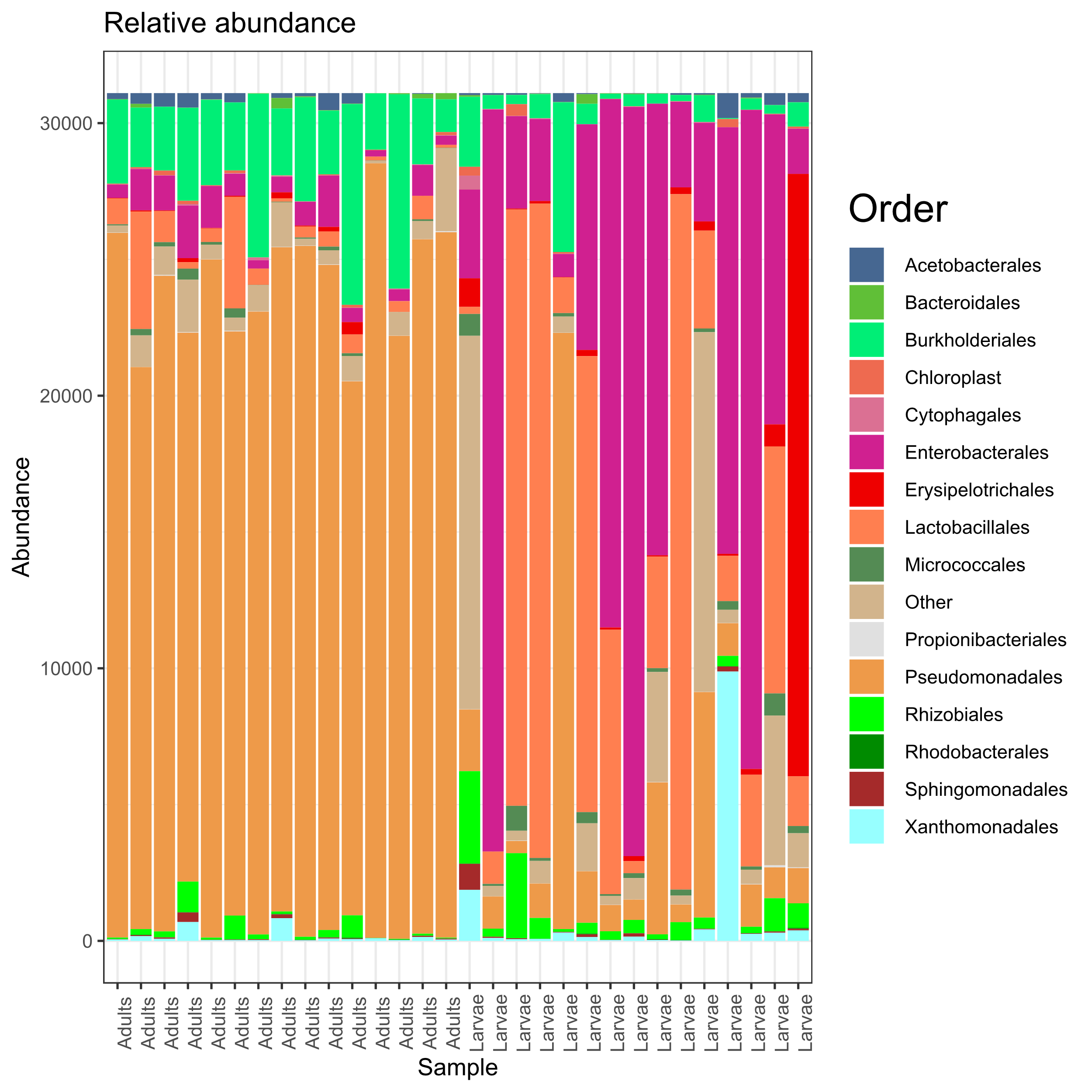
Supplementary Figure 2. Taxa Bar showing the top bacterial Order from both adult and larvae.


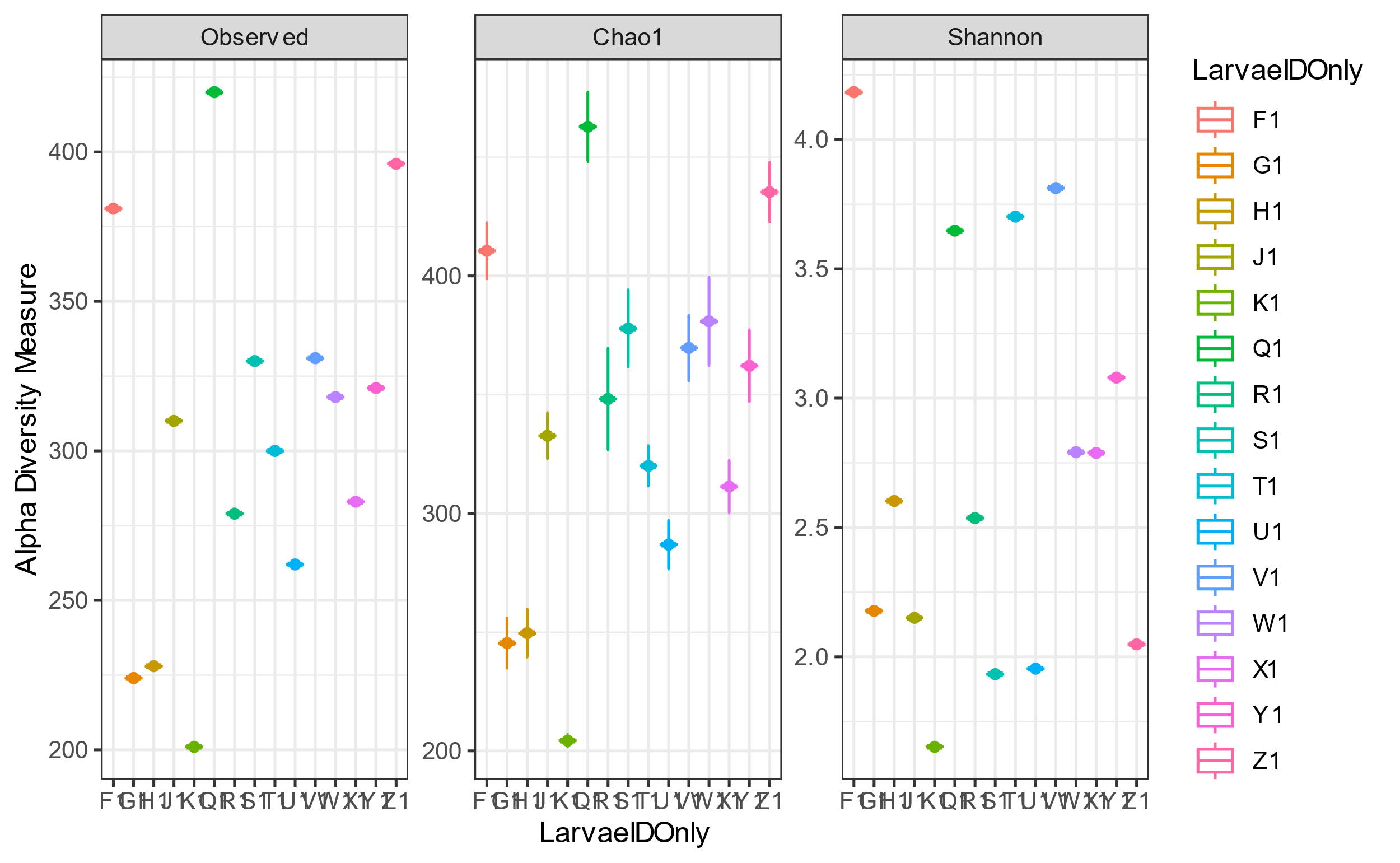
 Supplementary Figure 4. Alpha diversity indices-Observed, Chao1 andShannon of the larval samples.


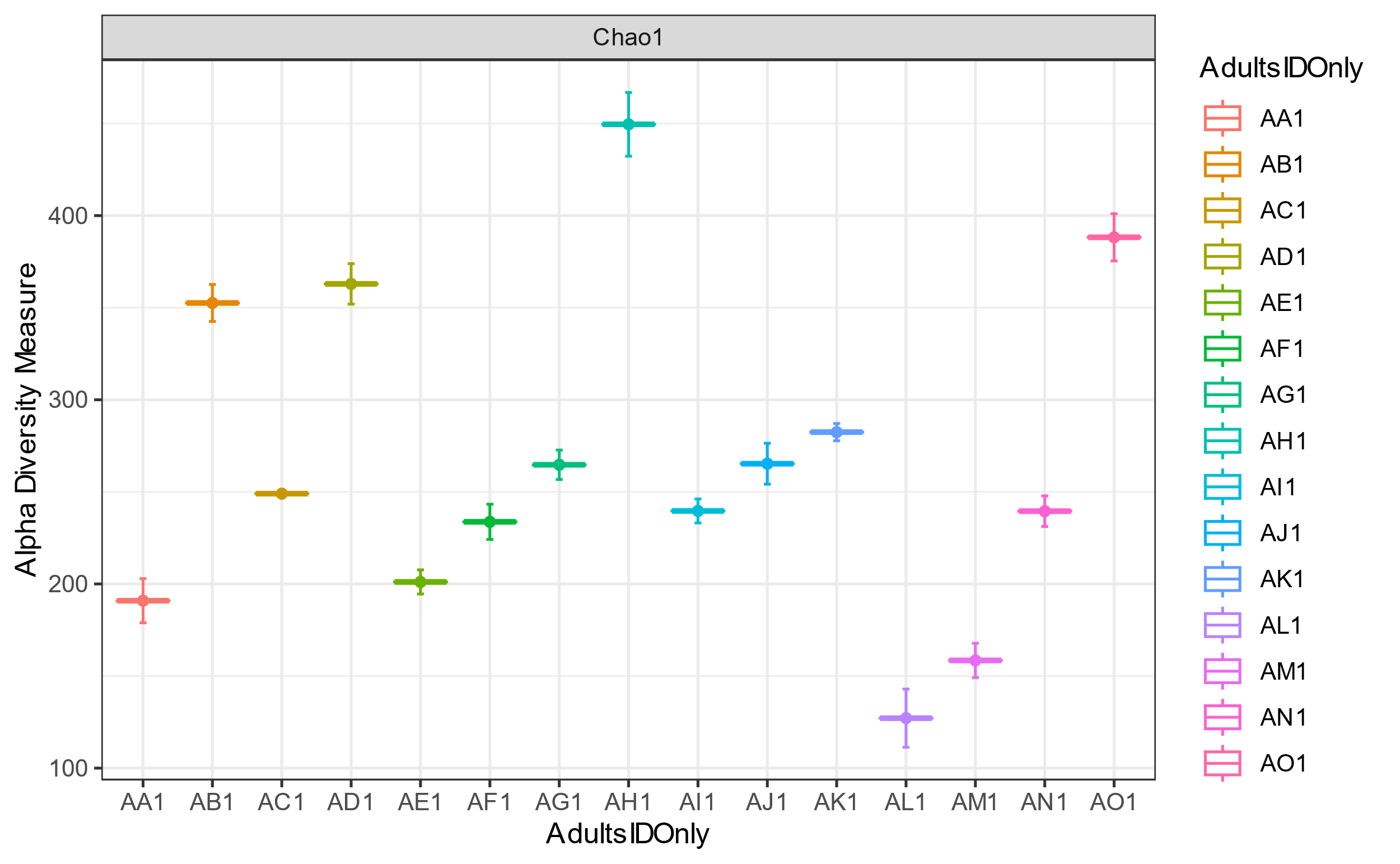
 Supplementary Figure 5. Alpha diversity indices-Observed, Chao1, Shannon and Simpson of the adult samples.
